# Estimating the volume of the vitrectomized space using axial length: a guideline

**DOI:** 10.5935/0004-2749.2024-0229

**Published:** 2024-12-26

**Authors:** Rodrigo Pessoa Cavalcanti Lira, Andrea Andrade Azevedo de Vasconcelos, Valesca Castro Neri, Juliana Moreira de Santana, Luiz Felipe Lynch de Moraes, Gabriel Rocha Lira, Maria Isabel Lynch Gaete

**Affiliations:** 1 Universidade Federal de Pernambuco, Recife, PE, Brazil

**Keywords:** Cataract extraction, Retinal perforations/surgery, Epiretinal membrane/surgery, Vitreous body, Axial length, eye, Vitrectomy, Biometry/methods, Diagnostic techniques, ophthalmological, Guidelines as topic

## Abstract

**Purpose:**

The volume of the vitreous chamber varies with the size of the eye. The space created in the vitreous cavity by a vitrectomy is called the vitrectomized space. The volume of the vitrectomized space is strongly correlated with the axial length of the eye. This study aims to present guidelines for estimating the using participants stratified by axial length, sex, and history of cataract surgery.

**Methods:**

This retrospective, observational, cross-sectional study included 144 randomly selected participants who underwent vitrectomies between 2013 and 2023. Before surgery, the axial lengths of participants' eyes were measured using optical biometrics. The axial lengths of the eyes in our sample were between 20-32 mm. In all cases, a complete vitrectomy was performed, followed by complete fluid-air exchange and injection of a balanced saline solution. The volume infused was recorded.

**Results:**

The median (interquartile range; range) volume of the vitrectomized space was 6.1 (3.8; 3.1-11.3) mL in men and 6.1 (3.3; 3.2-11.2) mL in women (p = 0.811). The median volume of the vitrectomized space was 5.9 (3.6; 3.1-11.2) mL in patients with phakic lenses and 6.25 (3.6; 3.3-11.3) mL in those with pseudophakic lenses (p=0.533). A positive correlation was found between the axial length and the volume of the vitrectomized space in this sample (r=0.968; p<0.001). In a cubic polynomial regression, the coefficient of determination was 0.948. Similar results were observed in both sexes and in both phakic and pseudophakic patients. The estimated cubic polynomial regression equation for this sample was

$$\text{VVS}=0.000589052857847605\times {\text{AL}}^{3}-0.025114926401582700\times {\text{AL}}^{2}+0.685961117595624000\times \text{AL}-5.088226672620790000$$

**Conclusion:**

We developed this axial length estimation of the volume of vitrectomized space as a guideline for the determination of vitrectomized space volume using axial length.

## INTRODUCTION

The vitreous chamber is the largest structure in the eye but varies in volume between individuals^(1^,^2^,^3)^. Several intraocular drugs can already be applied to this site, and there is an increasing number of new therapeutic agents designed for injection or implantation in the vitreous cavity^(4)^. Information on vitreous volume is necessary to understand the behavior of these agents once they are deposited in the vitreous^(5)^.

Azhdam et al.^(6)^ evaluated 100 eyes using highresolution computed tomography (CT). They found an average vitreous cavity volume (VCV) of 4.65 (±0.47) mL in women and 4.97 (±0.46) mL in men, with a positive correlation between axial length (AL) and VCV. They also demonstrated that the mean VCV is greater than the previously estimated 4 mL. A recent retrospective study used magnetic resonance imaging (MRI) scans from 72 eyes to develop a formula for the calculation of vitreous volume^(7)^.

Vitrectomized volume space (VVS) is a recent concept that describes the space created in the vitreous cavity by vitrectomy. This space is generally slightly smaller than the VCV as there is usually some residual vitreous after vitrectomy. Following a vitrectomy, the VVS is filled with a vitreous substitute (gas, perfluorocarbon liquids, silicone oil, natural polymers, gels, or hydrogels)^(8)^.

Tanaka et al.^(8)^ performed VVS volume measurements during the phacovitrectomies of 156 myopic individuals (average AL>26 mm) with retinal detachment to calculate the amount of 100% sulfur hexafluoride 6 (SF_6_) required to achieve the target concentration (15%) in the vitreous cavity. In another study, VVS measurements were taken from 114 individuals with pseudophakic lenses (26 mm > AL >21 mm) with macular holes or epiretinal membranes^(9)^. Again, both of these studies identified a strong positive correlation between AL and VVS (p<0.01).

Determining the relationship between VVS and AL may be useful in clinical research as it could increase dosage precision when administering intravitreal pharmacological agents, such as antibiotics and chemotherapeutic drugs, and vitreous substitutes after vitrectomy. This study aimed to develop guidelines for the estimation of VVS using Als.

## METHODS

### Patients

This retrospective, observational, cross-sectional study included individuals who underwent vitrectomy surgery for macular holes, epiretinal membranes, or vitreous opacities between 2013 and 2023 in Recife, Brazil. The study was approved by the Research Ethics Committee of the Clinical Hospital of the Federal University of Pernambuco (UFPE) (CAAE no. 26684819.4.0000.8807). All participants signed an informed consent form after receiving relevant information on the risks and benefits of their surgical procedure.

The inclusion criteria were an age >21 years and eyes with an AL between 20-32 mm. The exclusion criteria were a history of other vitreoretinal surgeries, cataract surgery complications that compromised the intraocular lens position, and other vitreoretinal diseases. From the 368 individuals identified who met these criteria, 144 were randomly selected and stratified by AL, sex, and history of cataract surgery. The participants were grouped into blocks of 12 for each 1 mm interval of AL (six phakic and six pseudophakic, with three males and three females in each subgroup).

### Procedures

Before surgery, the AL of each eye was measured using optical biometrics (IOLMaster^®^, Carl Zeiss^®^, Germany). All patients underwent the same surgical procedure, which was performed by the same surgeon. This consisted of complete pars plana vitrectomy with vitreous base scraping (Constellation^®^ vitrectomy system, Alcon Laboratories, USA). This was done using 23- or 25-gauge vitrectomy probes, three ports, and valved trocars inserted into the sclera 3.5-4 mm from the limbus. A complete air-fluid exchange was performed. Then, the air infusion pressure was adjusted to 10 mmHg to maintain the ocular volume, with air injected through the inferior temporal trocar. Balanced saline solution was injected through the superior temporal trocar using a 10 mL syringe in 0.2 mL increments (BD Medical^®^, Brazil) and/ or a 1 mL syringe in 0.1 mL increments (BD Medical^®^). During the final phase of filling the vitreous cavity with the solution, the trocars were carefully monitored for leaks, and the air infusion tube was temporarily closed to avoid reflux. The volume infused was recorded in each case.

### Statistical analyses

The Shapiro-Wilk test was used to evaluate the normality of continuous data distributions. Normally distributed variables were expressed as means and standard deviations (SDs). Non-normally distributed variables were expressed as medians and interquartile ranges (IQRs). Between-group differences in independent continuous variables were compared using the student's t-test with normally distributed data and the Mann-Whitney U test for non-normal distributions. With continuous, non-normally distributed variables, between-group comparisons were made using the Wilcoxon signed rank test. The correlation between AL (the independent variable) and VVS (the dependent variable) was calculated using Pearson's linear correlation coefficient. The coefficient of determination (R^2^) was also calculated. A cubic polynomial regression was conducted to fit the equation:

$$\text{Y}=\text{A}+\text{BX}+{\text{CX}}^{2}+{\text{DX}}^{3}$$

where Y is the VVS of the eye, X is the AL, and A, B, C, and D are the coefficient constants. All statistical analyses were performed using SPSS for Windows, version 21 (IBM Corp., Armonk, NY, USA). The p-values were bilateral, and statistical significance was set at p<0.05.

## RESULTS

The cohort in this study comprised 144 individuals who underwent vitrectomy surgery during the study period. The median (IQR; range) participant age was 60 (14; 38-79) years. In 74 of the participants (51.4%), the right eye was vitrectomized, in the remaining 70 (48.6%), the left eye. The median (IQR; range) AL was 25.93 (6.13; 20.01-31.99) mm (Table 1). The median (IQR; range) VVS was 6.1 (3.6; 3.1-11.3) mL. In men, the median (IQR; range) VVS was 6.1 (3.8; 3.1-11.3) mL; in women it was 6.1 (3.3; 3.2-11.2) mL (p=0.811). 1n participants with phakic lenses, the median VVS was 5.9 (3.6; 3.1-11.2) mL; in those with pseudophakic lenses, it was 6.25 (3.6; 3.3-11.3) mL (p = 0.533) (Table 2). In those who underwent surgery in the right eye, the median (IQR; range) VVS was 6.05 (39; 3.3-11.3) mL; in those who underwent surgery in the left eye, it was 6.3 (3.4; 3.1-11.2) mL (p = 0.577).

**Table 1:** Descriptive statistics for the axial lengths (mm) of study participants grouped by lens (phakic or Pseudophakic) and sex

	All participants			Phakic			Pseudophakic		
	All	M	F	All	M	F	All	M	F
No.	144	72	72	72	36	36	72	36	36
Mean	25.99	26.00	25.99	26.00	26.04	25.97	25.98	25.96	26.00
Median	25.93	25.93	25.87	25.89	25.89	25.95	25.93	25.93	25.87
SD	3.48	3.50	3.49	3.50	3.53	3.51	3.50	3.5	3.53
IQR	6.13	6.18	6.18	6.20	6.36	6.18	6.17	6.17	6.41
Range	11.98	11.94	11.86	11.98	11.79	11.74	11.82	11.71	11.72
Minimum	20.01	20.05	20.01	20.01	20.20	20.01	20.05	20.05	20.15
Maximum	31.99	31.99	31.87	31.99	31.99	31.75	31.87	31.76	31.87

IQR= interquartile range; F= female; M= male; N= number of participants; SD= standard deviation.

**Table 2:** Descriptive statistics for the volume of the vitrectomized space (mL) of study participants grouped by lens (phakic or pseudophakic) and sex

	All participants			Phakic			Pseudophakic		
	All	M	F	All	M	F	All	M	F
No.	144	72	72	72	36	36	72	36	36
Mean	6.4	6.5	6.3	6.3	6.3	6.2	6.5	6.6	6.4
Median	6.1	6.1	6.1	5.9	5.95	5.9	6.25	6.2	6.3
SD	2.1	2.2	2.0	2.1	2.2	2.0	2.1	2.3	2.0
IQR	3.6	3.8	3.3	3.6	3.9	3.2	3.6	3.8	3.6
Range	8.2	8.2	8.0	8.1	7.9	8.0	8.0	7.7	7.2
Minimum	3.1	3.1	3.2	3.1	3.1	3.2	3.3	3.6	3.3
Maximum	11.3	11.3	11.2	11.2	11.0	11.2	11.3	11.3	10.5

IQR= interquartile range; F= female; M= male; N= number of participants; SD= standard deviation.

A positive Pearson correlation coefficient (r=0.968; p<0.001) was found between AL and VVS. The cubic polynomial regression coefficient of determination was 0.948. Similar correlational results were observed in both sexes, and in both phakic and pseudophakic patients (Table 3). The estimated cubic polynomial regression equation for this sample was:

**Table 3:** Correlations between the axial length of the eye and the volume of the vitrectomized space in vitrectomy recipients and cubic polynomial regression

	Pearson's correlation coefficient	p	Cubic polynomial regression (R^2^)
All participants	0.968	<0.001	0.948
Phakic			
All	0.969	<0.001	0.950
Male	0.968	<0.001	0.954
Female	0.971	<0.001	0.955
Pseudophakic			
All	0.970	<0.001	0.951
Male	0.972	<0.001	0.964
Female	0.982	<0.001	0.966

AL= axial length of the eye; R^2^= coefficient of determination; VVS= volume of vitrectomized space.

**Figure 1:**
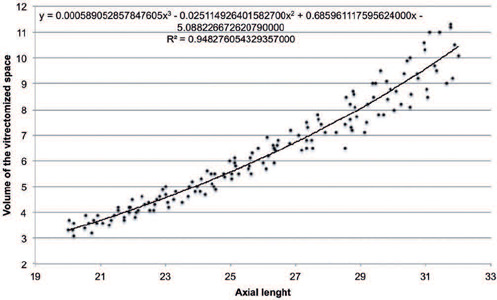
Correlations between the axial length of the eye and the volume of the vitrectomized space in vitrectomy recipients

$\text{VVS}=0.000589052857847605\times {\text{AL}}^{3}-0.0251149264015827\times {\text{AL}}^{2}+0.685961117595624\times \text{AL}-5.08822667262079$ (Figure 1).

We found no significant correlation between age and VVS (r=0.132; p = 0.116) or between age and AL (r=0.056; p=0.504).

Based on the above cubic polynomial equation derived from our in vivo data, Table 4 was compiled as a guideline for the determination of VVS using the AL. This lists AL values from 20 to 32 mm in 0.5 mm increments with the corresponding VVS for each. For each AL, the table provides a general VVS and the corresponding VVSs for patients with phakic and pseudophakic lenses. Thus, Table 4 constitutes our guideline for the use of **a**xial **l**ength to estimate the volume of **vi**trectomized **s**pace (ALVIS).

**Table 4:** Reference guidelines for using eye axial length to estimate the volume of the vitrectomized space (ALVIS)

Axial length (mm)	Volume of the vitrectomized space (mL)		
	General	Phakic	Pseudophakic
20.0	3.3	3.2	3.4
20.5	3.5	3.4	3.6
21.0	3.7	3.6	3.8
21.5	3.9	3.8	4.0
22.0	4.1	4.0	4.2
22.5	4.3	4.2	4.4
23.0	4.6	4.5	4.7
23.5	4.8	4.7	4.9
24.0	5.1	5.0	5.2
24.5	5.3	5.2	5.4
25.0	5.6	5.5	5.7
25.5	5.8	5.7	5.9
26.0	6.1	6.0	6.2
26.5	6.4	6.3	6.5
27.0	6.7	6.6	6.8
27.5	7.0	6.9	7.1
28.0	7.4	7.3	7.5
28.5	7.7	7.6	7.8
29.0	8.0	7.9	8.1
29.5	8.4	8.3	8.5
30.0	8.8	8.7	8.9
30.5	9.2	9.1	9.3
31.0	9.6	9.5	9.7
31.5	10.0	9.9	10.1
32.0	10.4	10.3	10.5

## DISCUSSION

Several previous studies have reported a correlation between eye AL and the VCV or VVS^(5^,^6^,^7^,^8^,^9)^. The results of the present study were concordant with the existing literature and showed his to be a sufficiently strong correlation to allow accurate VVS estimation from the AL value. As the correlation was positive, the VVS increased with increases in the AL. The coefficient of determination showed that more than 90% of VVS variation was attributable to AL (R²=0.968). The significant correlation between the VVS and AL is shown in the following equation:

$$\text{VVS}=0.000589052857847605\times {\text{AL}}^{3}-0.0251149264015827\times {\text{AL}}^{2}+0.685961117595624\times \text{AL}-5.08822667262079\left(\text{r}=0.968;\text{p}<0.001\right)$$

A number of previous studies have described other approaches to VCV and/ or VVS estimation. Azhdam et al. evaluated the VCV using CT images from 100 eyes with ALs between 22-27 mm^(6)^. Borkenstein et al. evaluated the VCV using MR1 scans of 72 eyes with ALs between 20-30 mm^(7)^. Tanaka et al. took intraoperative VVS measurements during the phacovitrectomies of 156 eyes with ALs between 26-30 mm^(8)^. This was achieved through measurement of the volume of fluid aspirated during fluid-air exchange. 1n the present study, our participants underwent vitrectomies and the volume infused into the vitreous chamber after air-fluid exchange was measured in 144 eyes with ALs between 20-32 mm. This covered a wider biometric range than these previous studies.

To allow a comparison between our volume estimation equation and those of the above three studies^(6^,^7^,^8)^, we used the data from eyes in the present study with ALs in the range of 26-27 mm as this biometric range was common to all four studies. We input the biometric data (AL) of these participants into our equation, as follows:

$$\text{Volume}=0.000589052857847605\times {\text{AL}}^{3}-0.0251149264015827\times {\text{AL}}^{2}+0.685961117595624\times \text{AL}-5.08822667262079$$

Using the relevant data from the present study, we also applied the equation obtained by Tanaka et al.^(8)^:

Volume=−9.29+0.60×AL , the equation obtained by Azhdam et al.^(6)^:

Volume=−4.2838+0.37493×AL , and an equation derived from the study by Borkenstein et al.^(7)^:

$$\text{Volume}=0.000140020398417651\times {\text{AL}}^{3}-0.00562156161649195\times {\text{AL}}^{2}+0.0823660330762208\times \text{AL}-1.66380687175514$$

According to the equation from the current study, the median (IQR) vitreous cavity volume of the cohort was 6.3 (0.2) mL. 6.5 (0.2) mL According to the equation of Tanaka et al.^(8)^, the median (IQR) vitreous cavity volume of the cohort was 6.5 (0.2) mL. According to the equation of Azhdam et al.^(6)^, the median (IQR) vitreous cavity volume of the cohort was 5.6 (0.1) mL. And, according to the equation derived from the study by Borkenstein et al.^(7)^, the median (IQR) vitreous cavity volume of the cohort was 7.0 (0.3) mL. Although not the main focus of this study, these data were somewhat surprising. By supposedly measuring the total volume of the vitreous cavity, it would be expected that the volumes obtained using the equation of Azhdam et al.^(6)^ would be greater than those from the other three. Since this was not the case, our comparison suggests that this computed tomography-derived equation underestimates volumes.

In the current study, we found no significant difference in VVS between men and women. This was contrary to the findings of Azhdam et al.^(6)^, Wong et al.^(10)^, and Shufelt et al.^(11)^, all of whom found the volume of the vitreous chamber to be slightly larger in men than in women. This was probably due to the smaller ALs observed in the non-stratified samples of these three studies. Although the study by Azhdam et al.^(6)^ indicated that VVS decreases with age, our results did not find age to be an important factor in the estimation of VVS.

The graph showing the correlation between AL and VVS suggests a nonlinear expansion of the vitreous chamber (Figure 1). Chau et al.^(12)^ showed that, despite their greater size, myopic eyes are not associated with a larger orbit. Therefore, the altered shape of myopic eyes may be due to the anatomical restriction imposed by the orbital bone walls^(13)^. Wen et al.^(14)^ showed that, although the AL, horizontal length, vertical length, and volume of highly myopic eyes are greater than those of emmetropic eyes, there is a greater increase in myopia of the AL than of the vertical and horizontal lengths. The shape of nonmyopic eyes is described by the global expansion model, while the shape of myopic eyes is described by the axial elongation model^(15)^. Atchison et al.^(13)^ have shown that the vitreous cavities of eyes with staphyloma have lower volumes than those of eyes with equatorial stretching or global expansion. This irregular expansion of the vitreous chamber and the three-dimensional nature of volume measurement likely explain the greater suitability of cubic polynomial than linear regression formulas to volume estimation.

A limitation of this study was our evaluation of VVS, which corresponds to the void in the vitreous chamber produced by air-fluid exchange after vitrectomy, rather than VCV^(8)^. This corresponds to the VCV deducted from the residual vitreous volume after vitrectomy. which we based on the difference between the ALVIS guideline table and the VIVEX formula table, we estimate that this represents around 5%-10% of the VCV^(7)^. Another limitation is that the ALVIS table does not include extreme biometric values (AL<20 mm or AL>32 mm); therefore, it is not applicable to patients with nanophthalmos or very young children.

This study also had some strengths. Among these was the wide ocular size range included in our sample (20-32 mm), both for phakic and pseudophakic individuals, and for both sexes. Although the pseudophakics presented a median VVS approximately 0.2-0.3 mL greater than that of the phakics, the difference between the groups was not significant. 1n phakic patients, the lens is, on average, 4 mm thick and 8.9 mm in diameter, while the intraocular lens is less than half that thickness, taking up less space in the anterior chamber and vitreous cavity^(16)^. The VVS was found to be similar between the right and left eyes.

This study broadens the discussion about the use of fixed doses of intravitreal drugs. Our findings suggest that we may be administering subtherapeutic doses to myopic patients and excessively high doses to hyperopic patients. Kazajkin and Ponomarev have presented a means of individualized dose estimation for use with intravitreal antibiotics to reduce the risk of toxic damage to the retina^(17)^. For example, the standard intravitreal dose of the antibiotic amikacin used to treat endophthalmitis is 0.4 mg^(18)^. Using the above dose estimation approach, for a hyperopic patient with an AL of 22 mm and a VVS of 4 mL, we would administer a dose of 0.10 mg/mL. However, for a myopic patient with an AL of 29 mm and a VVS of 8 mL, we would administer a dose of 0.05 mg/mL; 50% lower^(17)^. The same reasoning applies to other intravitreal drugs used to treat endophthalmitis such as vancomycin, cephalosporins, and antifungals, excessive doses of which are known to be toxic to the retina. Tanaka et al.^(8)^ have demonstrated that VVS measurements can also be useful in the adjustment of intravitreal gas measurements.

In this study, we have developed new guidelines for the determination of the VVS using the AL (ALVIS [axial length to estimate the volume of the vitrectomized space]). A within-subjects comparison of ALVIS VVS measurements with those obtained using imaging and those obtained during surgery in future research would help to establish the accuracy of this approach.
